# Electrochemical recognition of MMA-A biomarker for vitamin B_12_ deficiency based on β-cyclodextrin self-assembled on polyaminothiazole

**DOI:** 10.1039/d5ra03779d

**Published:** 2025-07-18

**Authors:** Anila Rose Cherian, Ditto Abraham Thadathil, Anitha Varghese

**Affiliations:** a Department of Chemistry, CHRIST (Deemed to be University) Bangalore Karnataka-560029 India anilarose.cherian@christuniversity.in

## Abstract

For the first time, the efficient electroreduction and identification of methyl methacrylate (MMA) was achieved using a carbon fiber paper (CFP) electrode modified with polyaminothiazole-β-cyclodextrin (PAT-β-CD). The recognition capabilities of the β-CD/PAT/CFP and PAT/CFP electrodes were investigated using cyclic voltammetry, revealing the significant influence of β-CD on the observed electroanalytical behaviour. Specifically, a PAT-β-CD modified CFP electrode was fabricated by electropolymerizing aminothiazole, serving as a substrate for β-CD self-assembly through hydrogen bonding between the hydroxyl groups of β-CD and the nitrogen atoms of the polyaminothiazole ring system. This as-prepared electrode exhibited a novel electrochemical method for the identification of MMA. Notably, the final β-CD/PAT/CFP electrode demonstrated superior electrocatalytic activity towards MMA reduction under optimized conditions compared to bare CFP and other modified electrodes. This modified electrode displayed an extended linear concentration range of 10 nM to 270 nM and a low limit of detection (LOD) of 0.6 nM. Furthermore, the electrocatalyst demonstrated excellent stability, repeatability, and negligible interference from other species. Finally, the developed β-CD/PAT/CFP electrode was successfully applied for the quantitative determination of MMA in human urine samples.

## Abstract 2

For the first time, the efficient electroreduction and identification of methyl methacrylate (MMA) was achieved using a carbon fiber paper (CFP) electrode modified with polyaminothiazole-β-cyclodextrin (PAT-β-CD).

## Graphical abstract

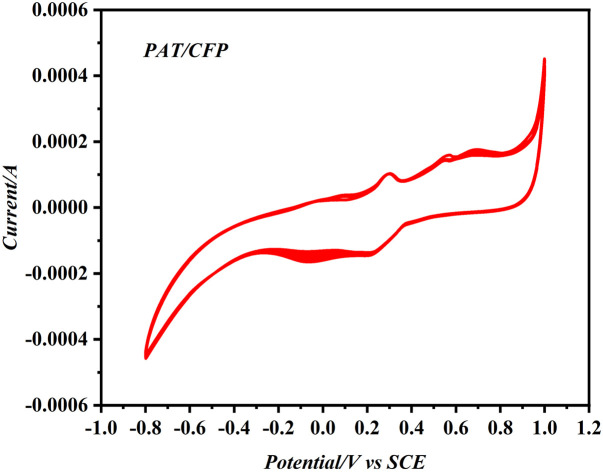

## 1 Introduction

Commonly referred to as cobalamin, vitamin B_12_ is a water-soluble vitamin that includes cobalt.vitamin B_12_ is required for DNA synthesis, red blood cell creation, and the maintenance of neurological functioning in the human body. It participates actively in human metabolism as two types of co-enzymes: cytosolic homocysteine methylation and mitochondrial l-methylmalonyl co-enzyme A conversion. Methylmalonyl CoA excess is transformed to methylmalonic acid (MMA). Vitamin B_12_ deficiency can result in pernicious anaemia, weariness, agitation, memory loss, decreased resistance to infection, and permanent and severe brain and nervous system damage. Serum vitamin B_12_ levels are commonly used to diagnose vitamin B_12_ deficiency; however, around 50% of people with subclinical illness have normal B_12_ levels. Serum MMA levels, which are elevated early in vitamin B_12_ insufficiency, offer a more sensitive technique of screening for vitamin B_12_ deficiency. As a result, understanding the need to create sensitive, reliable, and easy methods for determining vitamin B_12_ in medications and food a good availability of quantification methods are prevalent in literature.^1^ Small levels of MMA are required for human metabolism and energy generation. MMA is a dicarboxylic acid and *C*-methylated malonate derivative. If the body does not have an appropriate supply of vitamin B_12_, the concentration of MMA in the blood and urine rises. As a result, MMA is a distinct metabolic marker that is a more sensitive diagnostic of mild or severe vitamin B_12_ insufficiency.

Several methods have been reported for the quantification of MMA such as capillary electrophoresis with laser induced fluorescence,^2^ liquid chromatography-tandem mass spectroscopy,^3^ isotope dilution method,^4^ stable isotope dilution gas chromatography-mass chromatography,^5^ UPLC-MS/MS.^6^ These methods are disadvantageous as they are expensive, requires large amount of sample, complex sample preparation, heavy equipment, high consumption of reagents and buffers. These methods can be replaced by electrochemical sensors due to their several advantages such as low cost, high sensitivity and selectivity, fast response time, compatibility, simple modification, reproducibility, low power requirements and good stability.^7–11^ Electrochemical sensors for the detection of MMA converts the concentration of MMA in the blood serum or urine into an electrical signal.^12^

Polymer films modified electrodes have received a lot of attention in recent decades due to their wide range of applications in the domains of electrochemical sensors.^13^ Such modified films can greatly improve analyte electrocatalytic characteristics, increase reaction rate, and improve electrode response stability.^7,9^ Until recently, many methods for preparing polymer sheets modified electrodes have been employed, including coating, covalent bonding,^14–16^ and electro-polymerization.^17^ The electro-polymerization of organic compounds that contain beneficial functional groups (such as COOH, NH_2_, OH, SH, *etc.*) has demonstrated to be an effective technique for generating functionalized and electroactive polymers on electrodes, since the process can be readily managed by modifying the electrochemical parameters.^18^ As a result, the thickness, permeability, and charge transport properties of polymer-modified electrodes may be precisely specified, and the cost of sensors is kept cheap due to the ease of electro-deposition methods. Among the different monomers available 2-aminothiazole is able to undergo oxidative polymerization and results in the formation of a resilient adhesion on the substrate electrode, however in spite of it possessing diverse set of characteristics, reposts on the polymer, poly aminothiazole (PAT) preparations and its usage is limited. PAT can be modified with oxygen-containing groups such as hydroxyl, carboxyl, and epoxy groups, enhancing its hydrophilicity and biocompatibility. Cyclodextrins (CDs) are among these materials. Due to their primarily hydrophobic cavities of different sizes and various chemical modifications, CDs have been the subject of extensive electrochemical research, including both their behaviour in homogeneous solutions and thin films adhered to electrode surfaces.^19,20^ Through noncovalent interactions, host molecules may physically tolerate a variety of guests in host–guest systems. There are several synthetic receptors that operate as host molecules and feature a cage-like supramolecular structure. Because of their low toxicity and widespread availability, CDs are among the most commonly utilised hosts. Cyclodextrins (CDs) are a kind of natural cyclic oligosaccharides made of 6, 7, or 8 d-glucose units connected by 1,4-glycosidic linkages and denoted by the letters α-CD, β-CD, or γ-CD. Cyclodextrin has a torus-shaped physical structure with a hydrophilic perimeter and a somewhat hydrophobic centre.

Given the particular benefits of the electrochemical technique for precise control over redox processes, we propose that electrochemical oxidative polymerization of 2-aminothiazole (PAT) a way for modifying the surface characteristics of the substrate electrode, carbon fiber paper electrode (CFP) in our case. On the other hand very good sensing properties of cyclodextrins prompted us to investigate the potential function of beta-cyclodextrins (β-CD). As a result incorporating β-CD with PAT can provide an electrically conductive coating that boosts faradaic current. PAT's excellent qualities and the benefits of CDs have gotten increased attention in the development of high performance electrode materials. For these reasons, we decided to investigate the feasibility of employing CD/PAT/CFP as a platform for developing electrochemical sensors. The electrochemical identification of MMA molecules with β-CD (CD) self-assembled on poly(2-aminothiazole) (PAT) *via* its primary hydroxyl groups ((HO)p) is described in this work. Differential pulse voltammetry (DPV) is used to assess the chemical recognition capacity of the finally modified CD/PAT/CFP towards MMA, demonstrating a higher recognition ability for MMA.

## 2 Experimental

### 2.1 Reagents

Methylmalonic acid (CH_3_CH(COOH)_2_, 99%), 3 aminothiozole, sodium dodecylsulfate (SDS), silver nitrate (AgNO_3_) were purchased from Sigma Aldrich.

### 2.2 Instruments for characterization

In a three electrode, one-compartment glass cell, electrochemical polymerization and characterization were performed and this was connected to an electrochemical analyzer CHI608E (CH Instrument Inc. USA). The working electrode was a CFP electrode and platinum electrode is employed as an auxillary electrode. It is with respect to the saturated calomel electrode (SCE), the cell potentials are measured. All experiments were conducted at a temperature of 24 ± 3 °C. A FEI scanning electron microscope (model SIRION) was employed to capture SEM images. These were recorded using X-ray with a wavelength of *λ* = 1.5406 Å. The FTIR spectra were obtained utilizing a Thermo Nicolet Avatar 370. X-ray photoelectron spectroscopy (XPS) measurements were carried out with an Omicron ESCA probe spectrometer using polychromatic MgKα X-rays.

### 2.3 Development of β-cd self-assembled on PAT/CFP

The polymerization of aminothiazole (AT) was carried out in 0.2 M buffer solution (PBS, pH 7) comprising of 0.01 M AT in a three electrode cell employing a CFP as a working electrode, Pt foil as auxillary electrode and SCE was the reference electrode. Superimposed cyclic voltammograms (CV) were obtained between −0.8 V and 1.0 V at a scan rate of 50 mV s^−1^ over 15 cycles. Following this, the PAT/CFP (polyaminothiazole coated carbon fiber paper) was washed with distilled water and then immediately placed into a solution of 0.1 M PBS at pH 7, which contained 0.01 M β-CD, and allowed to soak overnight to facilitate the self-assembly of β-CD on the PAT/CFP. This process resulted in the creation of the modified CD/PAT/CFP electrode.

### 2.4 Preparation of human urine samples for real sample analysis

Urine samples from healthy individuals were gathered and stored in a refrigerator at 4 °C. Before analysis, the samples were diluted 100 times using PBS (0.2 M, pH 7.0) to reduce matrix complexity. Different amounts of MMA were added to the diluted urine samples. The amount of MMA at the modified electrodes, especially the CD/PAT/CFP electrode, was measured without any additional treatment steps. To evaluate the MMA levels, all measurements for both reference and real samples were conducted in the electrochemical cell following the proposed method.

## 3 Results and discussion

### 3.1 Electropolymerization of at followed by self-assembly of β-CD for the proposed sensor fabrication

**Fig. 1:**
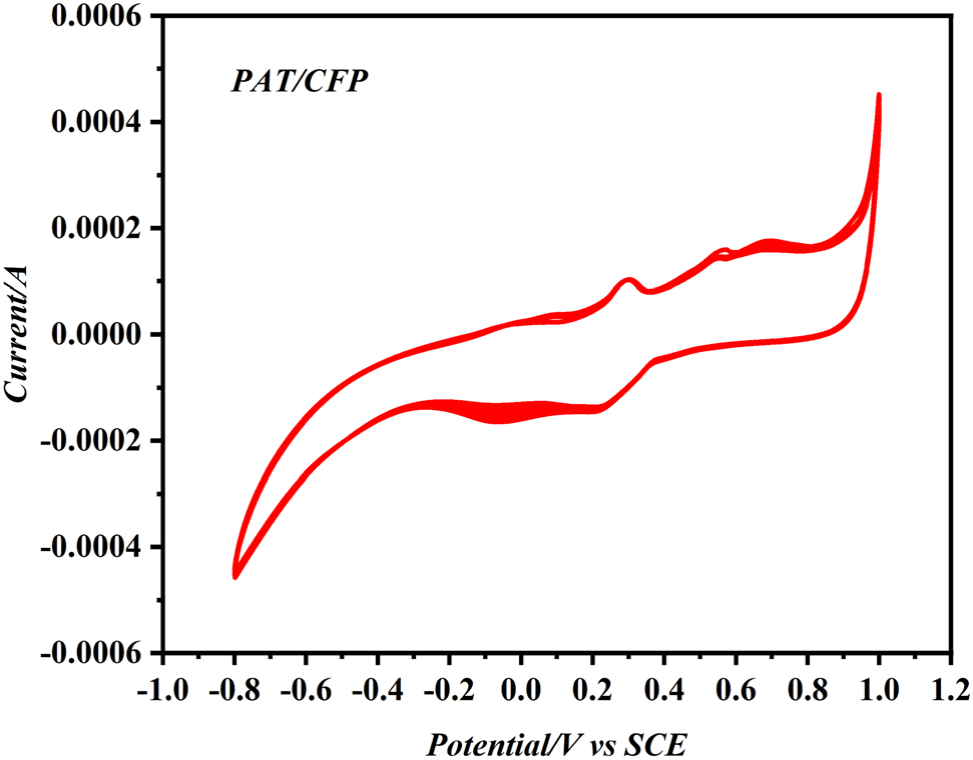
Voltammogram obtained for the electropolymerization of 0.01 M AT monomer in 0.2 M PBS on sweeping between potentials −1.0 V to 1.2 V.

The cyclic voltammograms (CVs) recorded during the electropolymerization of 0.01 M 2-aminothiazole (AT) in 0.2 M PBS are presented in Fig. 1. During the initial forward scan, a broad anodic peak appears at approximately 0.70 V, attributed to the oxidation of AT to its corresponding cation radical. A subsequent peak at ∼0.23 V corresponds to the coupling of the cation radical with the electrode surface, initiating polymer film formation. Unlike conventional electropolymerization processes where a gradual decrease in current is typically observed due to passivation or insulating film formation, our system exhibited a progressive increase in current over successive cycles. This behaviour is characteristic of conductive polymers such as polyaminothiazole (PAT), where the growing film enhances the electrode's electrochemical activity. The increase in current can be attributed to the continuous development of an electroactive PAT layer that provides additional redox-active sites and facilitates faster electron transfer. Similar behavior has been reported in other systems involving conductive polymer film growth, where the electrode becomes increasingly active as the polymer layer thickens.

**Scheme 1:**
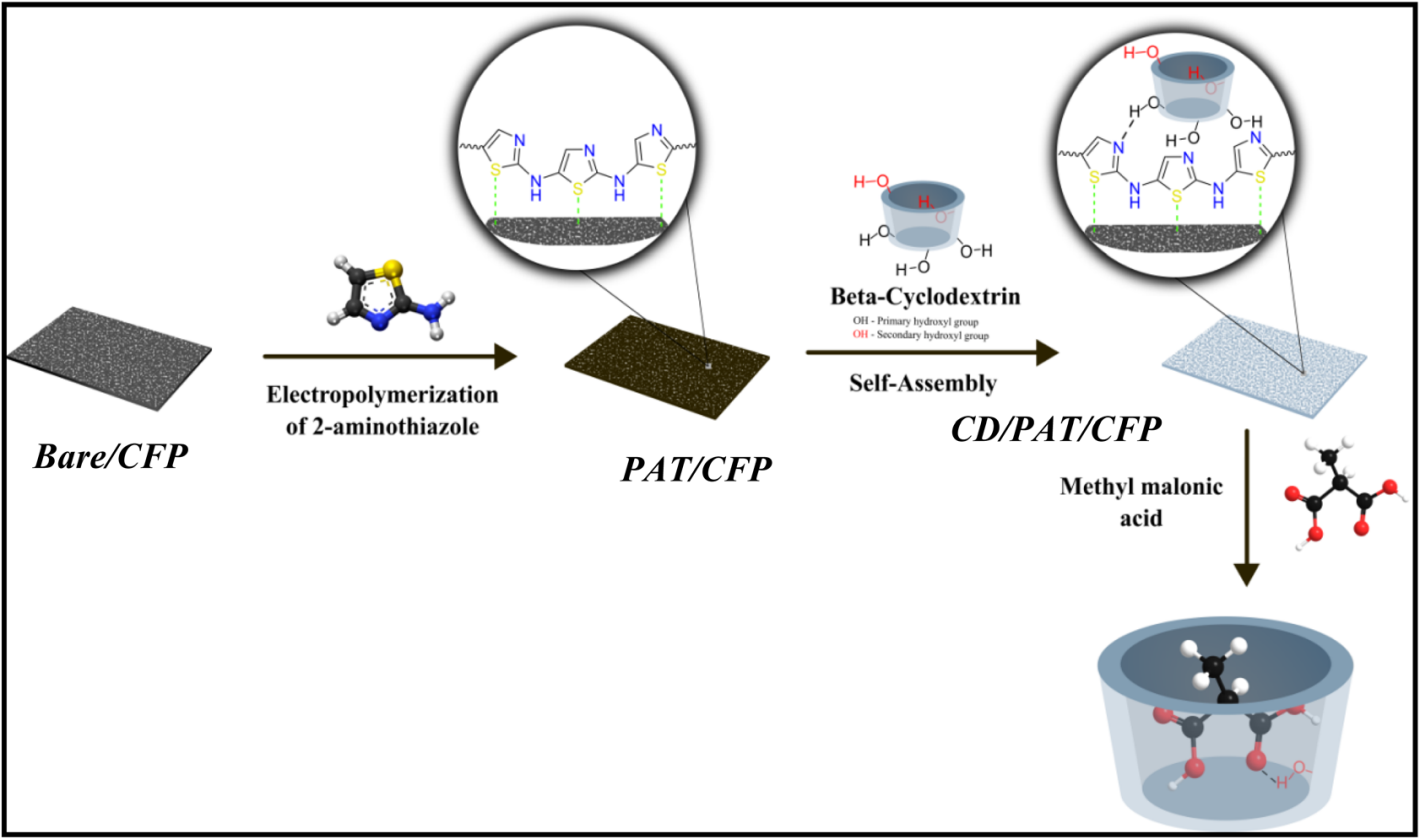
Depicts the different stages involved in the preparation of the MMA sensor.

Following polymer deposition, the PAT/CFP electrode was immersed in a 0.01 M β-cyclodextrin (β-CD) solution (in 0.1 M PBS, pH 7.0) and allowed to incubate overnight at room temperature to facilitate self-assembly. The β-CD molecules interact non-covalently with the PAT surface *via* hydrogen bonding between the primary hydroxyl groups of β-CD and the nitrogen atoms of the aminothiazole backbone. This results in the formation of the β-CD/PAT/CFP composite electrode, providing a supramolecular host–guest recognition interface for methylmalonic acid (MMA) sensing. The entire fabrication strategy is schematically illustrated in Scheme 1.

### 3.2 Electrochemical characterization of the bare and modified electrodes *via* CV and EIS

**Fig. 2:**
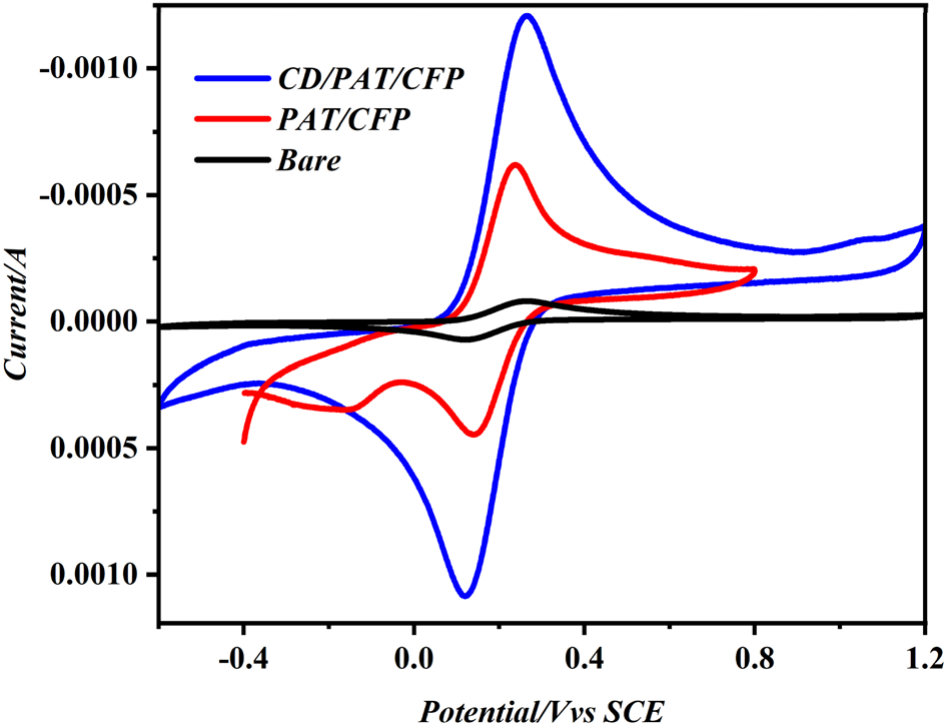
CV depicting the electrochemical response of bare/CFP, PAT/CFP and CD/PAT/CFP towards the redox probe in 0.1 M KCl.

Investigation of the electrochemical property of the modified electrodes was carried out by CV and EIS using 0.1 M KCl containing 2 mM of the electroactive redox probe (Fe(CN)_6_^4−/3−^) As displayed from Fig. 2, one can but notice redox peaks of the probe are well defined at the bare and modified CFP electrodes of Δ*E*_p_ values 150 mV for the bare/CFP, 137 mV for PAT/CFP and 114 mV for the CD/PAT/CFP electrode. We can see that the response improved on employing the modification and the Δ*E*_p_ value became relatively small. This is an indication of the modifications employed act as a conducting bridge that facilitates the transfer of electrons of the redox probe which in turn accelerates the redox process. Also, electron impedance spectroscopy (EIS) technique was carried out to gauge the electrical conducting ability of the different modified electrodes. Electrochemical impedance spectroscopy (EIS) serves as an effective method for examining the interfacial properties of electrodes that have been modified at their surfaces. This technique was also employed to illustrate the incremental construction process of the altered electrodes, with results consistent with earlier characterization through cyclic voltammetry (CV). The EIS results were analyzed using a corresponding circuit model. The high-frequency region observed in the Nyquist plot distinctly highlighted the series resistive behavior of the circuit components. Furthermore, the low-frequency region of the Nyquist plot accounted for the diffusion contributions by including the Warburg element in the equivalent circuit.

**Fig. 3:**
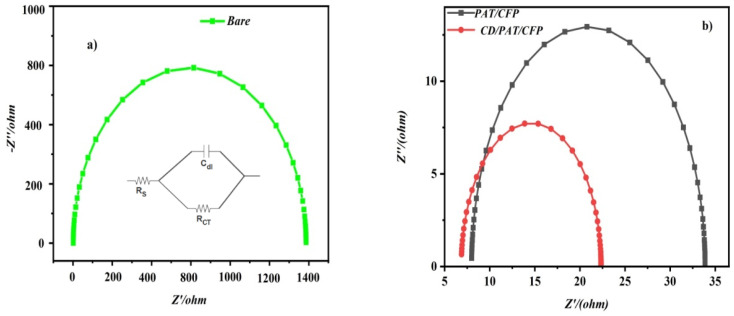
(a) Nyquist plots of bare CFP electrode (b) PAT/CFP electrode and CD/PAT/CFP electrode in 2 mM [Fe(CN)_6_^3−/4−^] in 0.1 M KCl.

From the Nyquist plots obtained Fig. 3a and b that were fitted with a simple Randles circuit, the charge transfer resistance values of the bare/CFP, PAT/CFP and CD/PAT/CFP electrode were 1400 Ω, 33 Ω and 22 Ω. The result revealed that CD/PAT/CFP electrode exhibited the smallest semi-circle with the lowest *R*_CT_ thereby having lower impedance than the other electrodes. The results show that the modifiers contributed improved conductivity and high specific-surface-area, which enhanced MMA reduction. The CDPAT/CFP interface acts as a modifier to boost the MMA probe's electron conduction channel. We validated the effective development of a thin coating on the CFP surface using EIS experiments.

Furthermore the coefficient of electron transfer (*k*_et_) was calculated from the below equation^21^*k*_et_ = *RT*/*n*^2^*F*^2^*AR*_CT_*C*where *n* is the number of electrons transferred of the redox probe, *C* is the concentration of the redox probe and the other symbols have their usual meaning. The hence calculated *k*_et_ are 2.5 × 10^−8^, 2.1 × 10^−6^ and 3.42.1 × 10^−6^ cm^2^ s^−1^ for the bare/CFP, PAT/CFP and CD/PAT/CFP electrodes respectively. The highest *k*_et_ values obtained for the CD/PAT/CFP electrode reveals that the process of electron transfer is the most facile at the surface of this electrode compared to the other electrodes.

### 3.3 Electrochemical behaviour of MMA at the surface of different electrodes

**Fig. 4:**
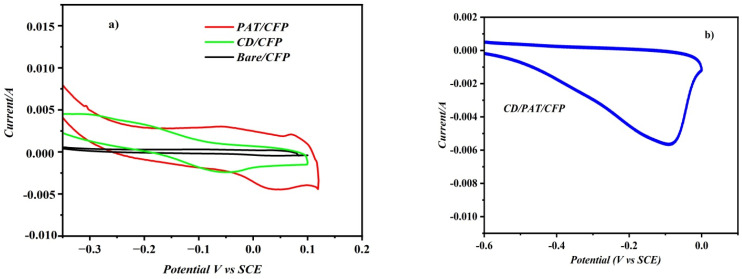
(a) CV responses of 75 nM MMA at PAT/CFP electrode, CD/CFP and bare CFP electrode in PBS (pH 7) between −0.4 V and 0.2 V *vs.* SCE at 0.05 V s^−1^. (b) CV response of CD/PAT/CFP Electrode in PBS (pH 7).

The performance of the CD/PAT/CFP electrode was assessed against unmodified CFP as well as other modified electrodes, including PAT/CFP and CD/CFP, to confirm its effectiveness for MMA detection. The experimental cyclic voltammetry (CV) results aligned well with those obtained from the electrochemical impedance spectroscopy (EIS) method. Comparison of the CVs of 75 nM MMA/0.1 M PBS (pH 7) at 50 mV s^−1^ recorded at the bare and modified working electrodes is displayed in Fig. 4. A poorly defined reduction peak of MMA is observed at the surface of the bare/CFP electrode. On comparing the electrochemical behaviour of MMA as seen in Fig. 4a at the PAT/CFP and CD/PAT/CFP we can observe that the reduction potential of MMA has shifted from 0.035 V (PAT/CFP) to −0.12 V (CD/PAT/CFP). A noticeable increase in the reduction peak current at the CD/PAT/CFP electrode ,as seen in Fig. 4b may be due to the synergistic effect of the aminothiazole polymer and the cyclodextrin moiety. On modifying the PAT/CFP electrode with self-assembled CD, it leads to the formation of a supramolecular host–guest interaction between the cyclodextrin moiety and the MMA molecules. The enhancement in the MMA reduction peak current at the CD/PAT/CFP electrode is attributed to the synergistic effect of the polyaminothiazole (PAT) polymer and β-cyclodextrin (β-CD). Although β-CD contains a hydrophobic inner cavity, inclusion complexation is not governed solely by hydrophobic interactions. Methylmalonic acid (MMA), being a hydrophilic dicarboxylic acid, can still interact with β-CD *via* hydrogen bonding and electrostatic interactions—particularly at the hydroxyl-rich rims of the β-CD cavity.

In the present system, β-CD is self-assembled onto the PAT backbone through hydrogen bonding between the hydroxyl groups of β-CD and the nitrogen atoms of the PAT ring. The carboxylic groups of MMA may form additional hydrogen bonds with both the β-CD rim and the functional groups in the PAT matrix. These non-covalent interactions result in an effective pre-concentration of MMA at the electrode surface, facilitating electron transfer. This is evidenced by the observed shift in the reduction potential and significant enhancement in reduction current in the presence of β-CD (Fig. 4).

**Fig. 5:**
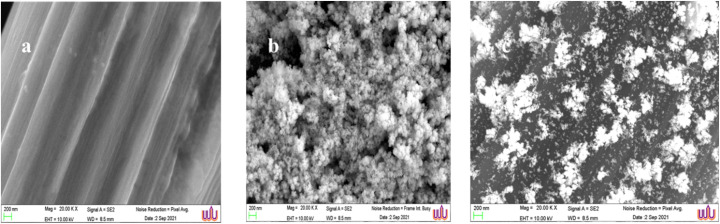
SEM images of (a) PAT/CFP, (b) CD/CFP electrode, (c) CD/PAT/CFP electrode.

The significantly enhanced reduction peak current observed at the CD/PAT/CFP electrode compared to bare CFP, PAT/CFP, and CD/CFP can be attributed to multiple complementary factors that support a plausible host–guest interaction between methylmalonic acid (MMA) and the β-cyclodextrin (β-CD) component:

• Synergistic effect: the noticeable increase in reduction peak current is attributed to the synergistic effect of both the polyaminothiazole (PAT) polymer and the β-cyclodextrin (β-CD) moiety. The PAT film provides a stable and conductive platform, while the self-assembled β-CD enhances the interaction with MMA.

• Electrostatic interaction and hydrogen bonding: the enhanced interaction stems from a combination of electrostatic interactions and hydrogen bonding. MMA, being a dicarboxylic acid, can form hydrogen bonds with the hydroxyl groups on the rim of the β-CD and with the nitrogen atoms of the PAT backbone.

• Concentration effect within the cavity: the presence of β-CD leads to a significant rise in the reduction signal of MMA, indicating that MMA penetrates into the relatively less polar cavity of the cyclodextrin moiety, thus leading to a concentration of the analyte at the electrode surface. This pre-concentration effect at the electrode surface facilitates the charge transfer process and results in a higher reduction current.

• Improved electron transfer kinetics: the EIS results Fig. 3a and calculated clearly demonstrate that the CD/PAT/CFP electrode exhibits significantly lower charge transfer resistance (*R*_CT_) and higher electron transfer coefficient compared to bare CFP and PAT/CFP electrodes. This indicates a more facile electron transfer process at the CD/PAT/CFP interface, which directly contributes to the observed increase in reduction peak current.

• Enhanced surface area: the FE-SEM images (Fig. 5c) reveal that the CD/PAT/CFP electrode possesses a highly porous surface compared to PAT/CFP. This increased electroactive surface area provides more active sites for the interaction with MMA and contributes to the enhanced current response.

The electrode current response rose successively, CD/PAT/CFP < PAT/CFP < CD/CFP < Bare CFP with indicating that the modifiers, when used separately, improved charge transit and definitely preferred MMA reduction. Based on the literature, the heightened peak signal may likely be attributed to its incorporation into the hydrophobic cavity of β-CD. This inclusion could lead to a concentration of the analyte at the electrode surface, influencing the kinetics of its charge transfer process. The small increase in the reduction peak current of MMA might be explained by a partial insertion of MMA into the β-CD. Conversely, the overvoltage associated with the MMA reduction reaction was lower, as *E*_p_ [CD/PAT/CFP] < *E*_p_ [PAT/CFP]. Notably, the slight reduction in overvoltage observed when CD/PAT/CFP is present clearly indicates its essential role in the electrocatalytic activity noted.

Although the CV profiles shown in Fig. 4b may appear less typical than textbook examples, their shape is consistent with the electrochemical behaviour of MMA under the specific experimental conditions employed in this study. These include a moderate scan rate, adsorption-controlled interaction at the electrode surface, and surface confinement due to the β-CD/PAT composite structure. The presence of well-defined reduction peaks, though slightly broad or asymmetric, clearly indicates the occurrence of MMA reduction. This interpretation has been clarified in the revised text to emphasize the peak potential shifts and current enhancements as indicators of electrode performance.

### 3.4 Characterization of the modified CFP electrodes by scanning electron microscopy

The surface morphology of the modified electrodes was scanned by FE-SEM. Fig. 5 displays the FE-SEM images of (a) PAT/CFP, (b) CD/CFP and (c) CD/PAT/CFP. The physical morphology of the different modifications can affect the electrochemical response of the prepared sensor. A very stark difference in the morphology was observed between PAT/CFP and CD/PAT/CFP as shown in Fig. 5a and c respectively. The PAT film on the substrate CFP electrode displays a uniform and smooth deposition of the polymer film. Whereas, on the other hand CD/PAT/CFP showcases a different morphology with a highly porous surface, thereby resulting in greater electroactive surface area, accelerated electron transfer and also better contact with the analyte, MMA resulting in higher and better catalytic activity.

### 3.5 Characterization of the modified CFP electrodes by X-ray photoelectron spectroscopy

**Fig. 6:**
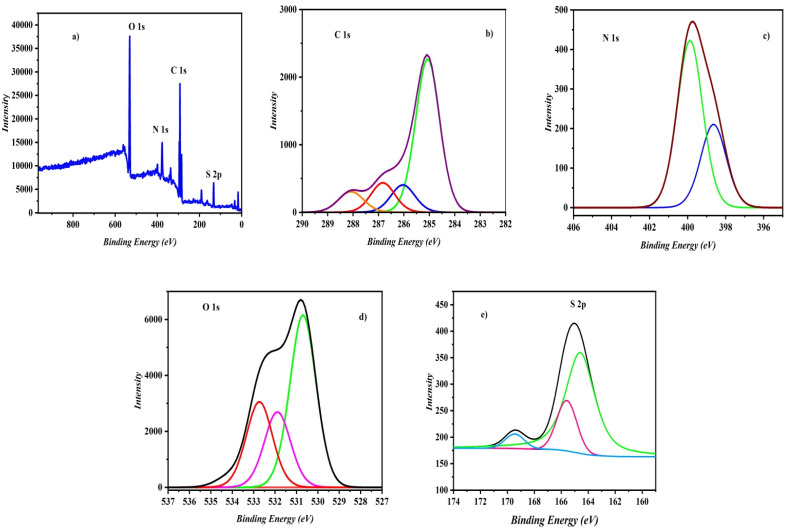
XPS studies of CD/PAT/CFP showing core level spectrum of (a) survey scan (b) C 1s spectrum, (c) N 1s spectrum, (d) O 1s spectrum and (e) S 2p spectrum.

XPS is an effective tool for analysing MIP electrodes. This analysis can provide insights into the composition, chemical state, and, notably, the binding energy of materials. XPS operates by illuminating a surface with X-rays, which generates photoelectrons. The energy of these emitted electrons correlates with their atomic and orbital sources. Consequently, XPS spectra convey details about the elemental composition of a surface.^22^ The XPS spectrum of CD/PAT/CFP is shown in Fig. 6. The deconvoluted high resolution C 1s spectrum as seen in Fig. 6b is divided into four peaks. A pronounced peak at 284.9 eV may be attributed to the enrichment of the C–C bond in carbon. The weaker signal at 286.04 eV confirms the presence of electron-poor carbon bonded to sulphur,^23^ while another weaker peak at 286.8 eV suggests the presence of a –N–C(S)=N– bond.^24^ At 288.1 eV, the existence C(O)–O bond associated with β-CD is shown by a peak. The N 1s XPS spectra, Fig. 6c decomposed into two peaks at 398.5 eV and 399.8 eV, indicating the presence of nitrogen atoms in amine (NH) and imine. Fig. 6d displays the deconvoluted spectrum for O 1s. Peaks for O=C–OH, C–O, and C–OH were found at 530.6 eV, 531.8 eV, and 532.7 eV, respectively.^25^ The sulphur binding in C–S bonds and conjugated –C=S– bonds can be ascribed to two distinct peaks in the XPS spectra of S 2p at binding energies of 164.0 eV (S 2p_3/2_) and 164.9 eV (S 2p_1/2_) as seen in Fig. 6e.

### 3.6 Kinetics behaviour of MMA at CD/PAT/CFP

**Fig. 7:**
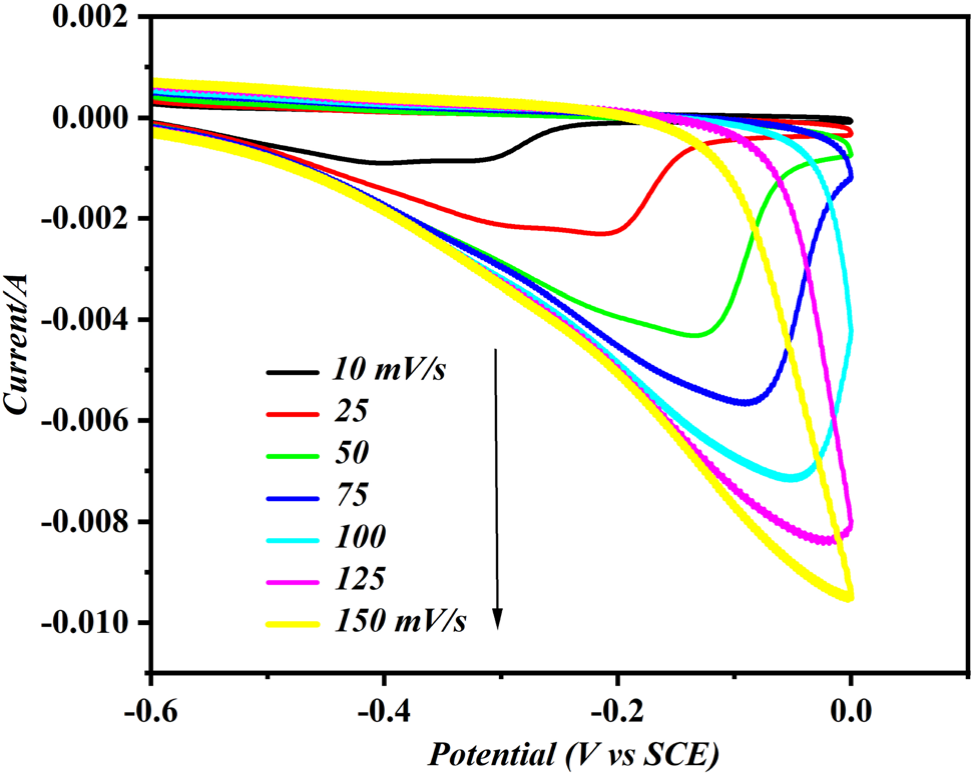
Superimposed cyclic voltammograms of 75 nM MMA at CD/PAT/CFP electrode at varying scan rates from 10 to 150 mV s^−1^ in PBS (pH 7).

Fig. 7 presents the overlaid cyclic voltammograms of MMA subjected to different scan rates, allowing for the assessment of whether the process occurring on CD/PAT/CFP is controlled by adsorption or diffusion. The reduction peak current for 75 nM MMA at the CD/PAT/CFP interface demonstrated excellent linearity as the scan rate increased from 10 mV s^−1^ to 250 mV s^−1^ in a supporting electrolyte of a buffer solution at pH 7, indicating that the process is likely adsorption controlled.^13^ This is also an indication the peak current is arising from the surface adsorbed MMA species. The relationship mention above can be established by the regression equation of*I*_p_ = −6.481 × 10^−4^*ν* −5.371 × 10^−4^, (*R*^2^ = 0.9884)

The total surface concentration (*Γ*) of electroactive MMA species on CD/PAT/CFP was then calculated using the equation *I* = *F*^2^*AΓn*^2^*n*/4*RT*^26^ where *A* is the total surface area and all the other symbols have their regular meaning. The computed surface concentration elevated in the order Bare CFP < CD/CFP < PAT/CFP < CD/PAT/CFP with values 9.65 × 10^−11^, 8.88 × 10^−9^, 22.96 × 10^−9^ and 45.32 × 10^−9^. These findings show that CD/PAT/CFP is capable of MMA reduction due to increased surface coverage concentration with HES.

Additionally, Bard and Faulkner^21^ noted that the electron-transfer coefficient, represented by *α*, can be calculated using the formula *α* = 47.7/(*E*_p_ − *E*_p/2_) mV. By inserting the values for *E*_p_ and *E*_p/2_, the electron transfer coefficient was determined to be 0.40. Furthermore, a strong linear correlation between peak potential and the logarithm of scan rate was observed, which can be described by the following equation.*E*_p_ (V) = 0.055 log *ν* (mV s^−1^) − 0.5772

Also, according to Laviron,^27^*E*_p_ is given by the following equation for an irreversible adsorption controlled process*E*_p_ = (2.303*RT*/(1 − *α*)*nF*)log *ν* + *K*Hence, for the electrochemical reduction of MMA the total number of electrons transferred was calculated to be 1.87, which is indicative of two electrons transferred during the process.

### 3.7 Effect of the solution pH

**Fig. 8:**
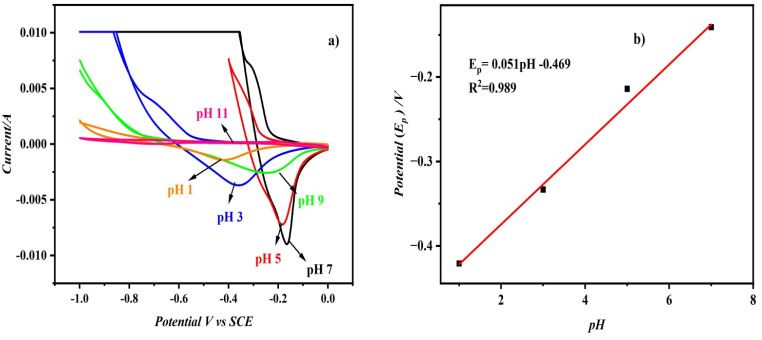
(a) CVs recorded using CD/PAT/CFP electrode for 75 nM MMA in PBS with pH ranging from 1–11. (b) pH *vs.* potential for MMA reduction.

Fig. 8a displays the superimposed cyclic voltammograms for 75 nM of MMA in pH solutions of pH 1, 3, 5, 7, 9 and 11. On increasing the solution pH, the reduction current increased as *I* = −0.0015*A* (pH 1.0), −0.0038 (pH 3.0), −0.0072 (pH 5.0), −0.0091 (pH 7.0), and notably the potential was shifted towards more positive potentials. However as the pH of the solutions increased to 9.0 and 11.0, the reduction current decreased, with the reduction peak potential shifting towards less positive potential. The decrease in the reduction current response maybe due to the electrostatic repulsion between deprotonated carboxylic acid functional groups on the surface of the modifier and the analyte. As a higher and pronounced reduction current peak was obtained for pH 7, further studies were carried out with the same pH. A linear relationship was established between the reduction peak potential of MMA against the pH of the electrolyte solution as in Fig. 8b. (*E*_P_ = 0.0512 pH − 0.469 with *R*^2^ = 0.9891). The measured slope value of 0.052 V pH^−1^ is quite near to the Nernst theoretical value of 59 mV pH^−1^, indicating that the reduction process is a two-electron process.

While the CVs recorded across different pH values (Fig. 8a) exhibit some deviations from ideal symmetry, they are characteristic of surface-confined, adsorption-mediated redox processes. The broad reduction peaks observed at neutral and mildly acidic conditions reflect the electrochemical response of MMA under pH-dependent interaction with the CD/PAT interface. The observed shift in peak potential with pH and the trend in current intensity support the occurrence of a proton-coupled electron transfer process. These results are in agreement with the electrochemical mechanism proposed for MMA reduction.

### 3.8 Sensitive determination of MMA using differential pulse voltammetry (DPV)

The DPV method utilizing minimal background current was employed to create a rapid and highly sensitive CD/PAT/CFP for measuring MMA. First, all operational parameters of the DPV were adjusted to enhance the sensitivity and precision for detecting trace amounts of MMA. We observed that with these optimized DPV configurations, the peaks were more distinctly defined at lower concentrations of MMA compared to CV.

**Fig. 9:**
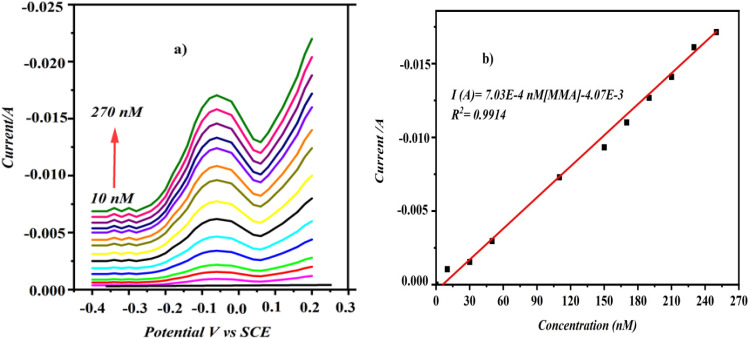
(a) DPV curves of MMA in PBS (pH-7) at CD/PATCFP electrode. (b) Plot of reduction current against concentration of MMA.

Fig. 9a shows DPV with well-defined curves about 340 mV, which corresponds to the MMA reduction process. The voltammograms show that MMA peak current values varied linearly on increasing the analyte concentration. The calibration plot of current against concentration of MMA is linear and is expressed as *I* (A) = 7.03 × 10^−4^ [MMA] − 4.07 × 10^−3^; *R*^2^ = 0.9914. The gradual increments of reduction current from the MMA measurements suggest that the modifications made to the CFP electrode are highly stable and responsive. The detection limit was determined using the formula LOD = 3*σ*/*S*, where *σ* represents the standard deviation of the peak current for a specific analyte concentration and *S* is the calibration plot slope.^28^ The LOD value for MMA at CD/PAT/CFP was furnished to be 0.6 nM. The advantage of the pulse method (DPV) is found in the differing decay rates of the charging current and faradaic currents. This enhanced ratio of faradaic current to capacitive current results in an excellent detection limit, suitable for precise and selective determination of MMA.

### 3.9 Selectivity and interference study

An interference analysis was performed to investigate the sensor's selectivity for MMA before assessing CD/PAT/CFP for identifying MMA in actual samples. Because we wanted to measure MMA in urine we used malonic acid (MA) as an interfering molecule. If one has increased methylmalonic acid levels as well as elevated malonic acid levels, one may have the metabolic illness, combined malonic and methylmalonic aciduria. The preliminary assessment of CD/PAT/CFP anti-interference features of by CV approach was studied.In this case, a binary combination of 75 nM MAA and 300 nM MA in PBS 7.5 was used. Even in the presence of such large quantities of MA, the initial CV research revealed no interference with the MMA current response. MA did not show a peak on CD/PAT/CFP during CV analysis. However, because the DPV approach lacks a strong capacitive current, a very small shoulder-like peak formed at −300 mV matching to MA during DPV analysis. MA, on the other hand, showed a peak at greater negative electrode potential but at a lower current density value, and it did not interfere up to a 900 nM concentration. Based on the findings, it is possible to conclude that the closely related compounds do not interfere with the determination of MMA when the suggested approach is applied for analysis. In the determination of MMA in actual samples, the suggested approach has a very high selectivity.

As possible interferent species, we conducted a selectivity investigation in the presence citric acid, l-glutamic acid and starch. The DPV was measured using a combination of these interferents and MMA in the electrochemical cell. We found that the sensitivity of MMA measurement remained consistent even with various organic interfering substances present. In another set of experiments, inorganic ions were tested as interferents. The maximum allowable concentration for interferents was determined to yield a relative error of less than 3% for a 75 nM MMA measurement. When the concentrations of Ca^2+^, Mg^2+^, K^+^, and Na^+^ were increased by a factor of 500, there was no interference observed in the quantitative analysis of MMA. These experiments confirmed the method's specificity, suggesting that the measurement of MMA in actual samples, such as urine, will remain unaffected by these common interfering substances.

### 3.10 The effect of the current sensor's stability and repeatability

**Fig. 10:**
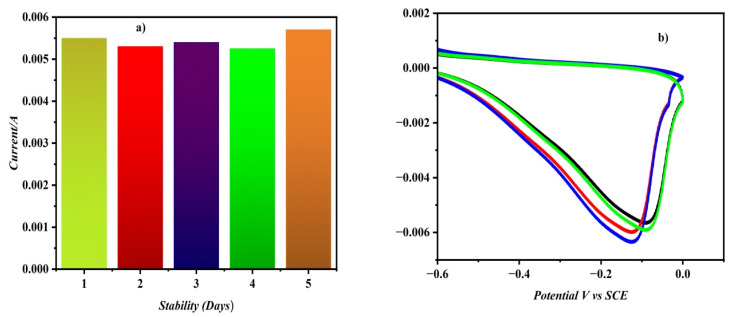
(a) Stability against reduction current bar graph profile (b). CVs for 75 nM MMA in 0 PBS at five distinct independent modified electrodes at a scan rate of 50 mV s^−1^.

The reproducibility of the electrode for measuring MMA was assessed by conducting the experiment four times with standard MMA solutions (75 nM), and the relative standard deviation (RSD) for the electrode's sensitivity to the MMA solution was found to be 1.4%, which is within acceptable limits. Additionally, the consistency of the electrode's response was further examined by using five modified electrodes for MMA measurement, resulting in an RSD of 1.7%. The fabricated electrodes demonstrated adequate repeatability and reproducibility in the measurement of MMA. Fig. 10a depicts a bar graph profile of MMA reduction currents *vs.* modified electrode stability (number of days). The obtained data clearly indicated that the nanocomposite electrode was stable. The repeatability of the current sensor was investigated by measuring the CV for 75 nM at five separate modified electrodes. The reduction currents obtained are nearly identical with a minor potential shift for all five distinct electrodes as seen in Fig. 10b.

### 3.11 Real sample analysis – practical application

The effectiveness of the proposed method was evaluated by analyzing MMA in human urine samples that had been artificially spiked. These samples were obtained from healthy individuals and processed following the sample preparation procedures described in the experimental section. The concentration of MMA in the samples was calculated using the standard addition technique and the DPV method. The spiked MMA samples were diluted with PBS (pH 7.0), and only the suitable amounts were used for measuring MMA levels. A reduction peak corresponding to MMA was observed in the differential pulse voltammograms of the urine samples. The results validate the proposed method's capability to effectively quantify MMA in real-world samples (Table 1). The levels of recovery were observed with minimum RSD values.

**Table 1:** Analysis of the analyte (MMA) in the spiked urine samples

Sample	*C*_added_ (nM)	*C*_expected_ (nM)	*C*_found_ a (nM)	Recovery (%)	RSD^a^ (%)
Urine sample 1	0	20.00	20.00	100.00	1.00
	2.5	22.50	22.40	99.55	1.12
	5.0	25.00	24.00	96.00	1.04
	10.0	30.00	29.50	98.83	0.87
Urine sample 2	0	30.00	29.00	96.66	0.98
	2.5	32.50	32.40	99.69	1.44
	5.0	35.00	34.50	98.57	1.02

a Mean value of five determinations; *c* is the concentration added.

### 3.12 Comparison with previously reported MMA detection methods

To highlight the performance of the proposed CD/PAT/CFP electrochemical sensor, a comparative analysis was performed against previously reported methods for methylmalonic acid (MMA) detection^1,29–32^. The results are summarized in Table 2, which lists the techniques, sensing platforms, linear concentration ranges, and corresponding limits of detection (LOD).

**Table 2:** Comparison of MMA detection methods reported in literature

Sl. No.	Method	Material/approach	Linear range	LOD	Reference
1	Electrochemical (PdAu-PPy/CFP)	PdAu-polypyrrole on CFP	4.01 pM to 52.5 nM	1.32 pM	1
2	Electrochemical	(Ag-PEDOT/PGE)	0.50 pM to 55 nM	(0.16 pM)	29
3	Electrochemical	Ti_3_C_2_T_*x*_-MXene/Fe_3_O_4_	9 × 10^−15^ mol L^−1^ to 9 × 10^−13^ mol L^−1^	2.33 × 10^−16^ mol L^−1^	30
4	Optical sensing	Multifunctional lanthanide MOFs	—	(1.07 × 10^−5^ M)	31
5	Optical sensing	Co based coordination polymer	—	—	32
6	Electrochemical (this work)	β-CD/PAT-modified carbon fiber paper	10–270 nM	0.6 nM	This work

## 4 Conclusions

The electroreduction and identification of MMA was effectively accomplished for the first time utilising a CFP electrode modified with polyaminothiazole—cyclodextrin. Using cyclic voltammetry, the recognition efficiency of CD/PAT/CFP and PAT/CFP was investigated. The results show that β-CD plays an important influence in the observed electroanalytical behaviour. In summary, we created a PAT/-CD-modified CFP by electropolymerizing aminothiazole as the substrate for β-CD self-assembly *via* hydrogen bonding between the (HO)p of β-CD and the N groups of ring system. At the as-prepared electrode, an intriguing phenomena of electrochemical identification of MMA is observed. When compared to bare CFP and other modified electrodes, the finally modified electrode, CD/PAT/CFP electrode demonstrated better electrocatalytic activity for the reduction of MMA under optimal circumstances. The CD/PAT/CFP demonstrated an extended linear range concentration of 10 nM to 270 nM and a LOD of 0.6 nM. It is a very stable and repeatable electrocatalyst with no interference. Finally, the current electrocatalyst was successfully used for quantitative MMA analysis in human urine samples.

## Ethical statement

All experiments were performed in accordance with the Guidelines for Human Sample Collection and Research of the Indian Council of Medical Research (ICMR), and experiments were approved by the ethics committee at Christ (Deemed to be) University. Informed consents were obtained from all human participants of this study.

## Author contributions

Anila Rose Cherian: conceptualization, data curation, formal analysis, investigation, methodology, writing – original draft, writing – review & editing. Ditto Abraham Thadathil: formal analysis. Anitha Varghese: supervision, validation, visualization.

## Conflicts of interest

There are no conflicts to declare.

## Data Availability

All data supporting the findings of this study are available within the article.
